# Protection efficacy and the safety of the synergy between modified Bazhen powder and PRRSV modified-live virus vaccine against HP-PRRSV in piglets

**DOI:** 10.3389/fvets.2024.1436426

**Published:** 2024-08-05

**Authors:** Hua Chai, Yanru Wei, Wenguang Chen, Guorui Han, Bello-Onaghise Godspower, Yanyan Liu, Chunliu Dong, Zhiyun Zhang, Yanhua Li

**Affiliations:** ^1^College of Veterinary Medicine, Northeast Agricultural University, Harbin, China; ^2^Department of Animal Science, Faculty of Agriculture, University of Benin, Benin City, Nigeria

**Keywords:** HP-PRRSV HuN4, modified live vaccine, modified Bazhen powder, efficacy, safety, synergy

## Abstract

The highly pathogenic porcine reproductive and respiratory syndrome virus (HP-PRRSV) poses a significant threat to the global swine industry. Vaccination is a preventive measure against viral infections. However, the use of vaccines in livestock healthcare programs faces the challenge of safety and delayed immune responses. Earlier studies have shown the potential of modified Bazhen powder as an immunomodulator with significant biological properties, but its effect on vaccines against HP-PRRSV is yet to be studied. This study elucidated how modified Bazhen powder could improve the safety and efficacy of the conventional PRRSV vaccine by evaluating T-cell responses, antibody levels, clinical symptoms, levels of viremia, organ health, and cytokine production. The results revealed that the oral application of modified Bazhen powder in combination with PRRS vaccination improved both cellular and humoral immunity, accelerated viremia clearance, improved lung injury scores, and reduced viral load in the tonsils. The modified Bazhen powder also effectively reduced inflammatory responses following a PRRSV challenge. These findings further highlight the pharmacological properties of modified Bazhen powder as a potential oral immunomodulatory agent that could enhance vaccine efficacy and ensure broad-spectrum protection against HP-PRRSV in pigs.

## Graphical abstract

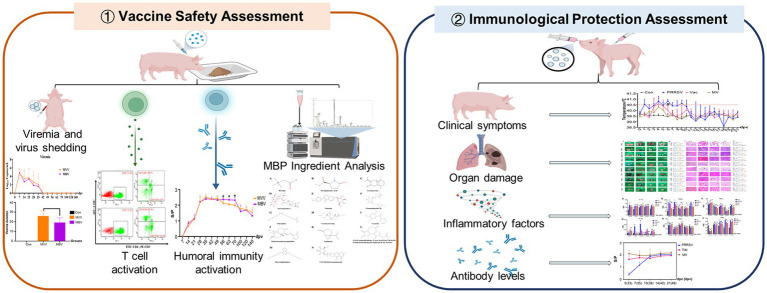

## 1 Introduction

Porcine reproductive and respiratory syndrome (PRRS) is a severe and highly contagious disease caused by the porcine reproductive and respiratory syndrome virus (PRRSV). Since its outbreak in the 1980s (1), PRRS has led to significant economic losses in the global swine industry. In 2006, China recorded an outbreak of the highly pathogenic PRRSV (HP-PRRSV) with high morbidity and mortality (2). The PRRSV strain HuN4, isolated from pig farms in the central region of China, has been identified as one of the HP-PRRSV strains responsible for more than 50% of PRRSV infections and 20 to 100% of deaths in pig farms (3).

Despite extensive research, a reliable treatment for PRRS remains elusive. Vaccination is critical in preventing PRRSV infection, with available options including inactivated and modified live vaccines (MLV). However, these vaccines often fail to provide comprehensive protection against the diverse strains found in the field and carry the risk of reverting to higher virulence (4). Moreover, commercial PRRSV MLVs can induce delayed and weak immune responses, including humoral and cell-mediated responses (5, 6). Although neutralizing antibodies (NA) are essential for fighting PRRSV, vaccination does not consistently result in high NA levels (7). Additionally, the risk of vaccine virus transmission to unvaccinated animals increases due to potential viremia and shedding post-vaccination (8). Hence, there is an urgent need to develop new strategies to improve the safety and efficacy of PRRSV vaccines for effective disease control.

Traditional Chinese medicine (TCM) offers a range of health benefits, including reducing inflammation, inhibiting viruses (4), and supporting the immune system. The immunomodulatory strategies of TCMs with anti-PRRSV effect involve the regulation of cytokines production in host cells and inhibiting pathways that promote PRSSV pathogenesis (4). Thus, combining immunomodulating TCMs with vaccines helps to boost the body’s defense system by increasing interferon expression and raising antibody levels (9–11). Based on this, we postulated that adding TCM, particularly modified Bazhen powder (MBP), to the PRRSV MLV vaccine could strengthen the immune response, attenuate PRRSV virulence, and lower the chances of transmission.

Bazhen, a traditional Chinese herbal prescription comprising eight commonly used herbs, is reported to possess immunomodulatory effects, enhancing T lymphocyte proliferation and IFN-γ secretion (12, 13). Furthermore, Bazhen has shown efficacy in reducing oxidative stress and inflammatory cytokines. Geng et al. showed that adding MBP to sows’ diets significantly enhanced production performance and improved health parameters in sows and weaned piglets (14). Despite these findings, the potential immunomodulatory effects of combining Bazhen with vaccines remain unexplored. This study elucidated how MBP combined with MLV can effectively control PRRSV prevalence in the swine industry.

To sum up, our study revealed how the oral administration of MBP in combination with PRRSV MLV enhanced the immune response to vaccination in growing pigs and protected them against the HP-PRRSV challenge. We also analyzed the active ingredients of MBP absorbed into the bloodstream. The mechanism behind the potential immunopotentiating effects of combining MBP with vaccines will provide new strategies for enhancing vaccine efficacy and control of PRRSV in the swine industry.

## 2 Materials and methods

### 2.1 MBP formula

All herbs in the formulation were obtained from the Chinese herbal market in Harbin (Harbin, China), all the plant materials were identified by Professor Yanyan Liu (College of Animal Medicine, Northeast Agricultural University, Harbin, China) and preserved in the Animal Pharmacy Laboratory of, Northeast Agricultural University. They contained Astragalus root (*Astragalus membranaceus*), Atractylodes rhizome (*Atractylodes lancea*), Hoelen (*Poria cocos Wolf*), Glycyrrhizae Radix (*Glycyrrihiza uralensis Fischer et DC.*), Raix Rubra (*Paeonia albiflora Pallas* var. *trichocarpa Bunge*), Angelica root (*Angelica sinensis*), Rehmanniae Radix et Rhizoma (*Rehmannia glutinosa Liboschitz*), *Ziziphus jujuba* Mill (*Ziziphus zizyphus*), Ligustici Chuanxiong Rhizoma (*Ligusticum chuanxiong Hortorum*) and Maltiflower Knotweed (*Fallopia multiflora (Thunb.) Harald*).

### 2.2 Viral strains and cells

The commercial live attenuated vaccine HuN4-F112 and the HP-PRRSV HuN4 strain (GenBank accession number: EF635006) were provided by Professor Tian from Harbin Veterinary Research Institute, Chinese Academy of Agricultural Sciences. Marc-145 cells used in this study were obtained from the stocks of Harbin Pharmaceutical Group Bio-vaccine Co., Ltd. Marc-145 cells, a PRRSV-permissive cell line derived from the African green monkey kidney cell line MA-104, grown in Dulbecco’s minimum essential medium (DMEM, Gibco) supplemented with 10% fetal bovine serum (FBS, CLARK) and 100 IU/mL penicillin and 100 μg/mL streptomycin at 37°C with 5% CO_2_.

### 2.3 Ethics approval

All animal experiments were done following protocols approved by the Experimental Animal Ethical Committee of Northeast Agricultural University (NEAUEC202303113). Animals were housed in environmentally conducive and specific pathogen-free laboratories. They were fed *ad libitum* with commercial feed and had unrestricted access to water. They were made to acclimatize to the environment for at least one week before the experiment. The animals were not subjected to stress during the experiment.

### 2.4 Animals

A total of 30 landrace piglets (four weeks old) were housed in the animal house of the Harbin Pharmaceutical Group Bio-vaccine Co., Ltd. The piglets were confirmed to be free of PRRSV, PRV, PCV, and Mph. They were provided with adequate water and a standard diet throughout the study.

### 2.5 Experimental procedures

**Figure 1:**
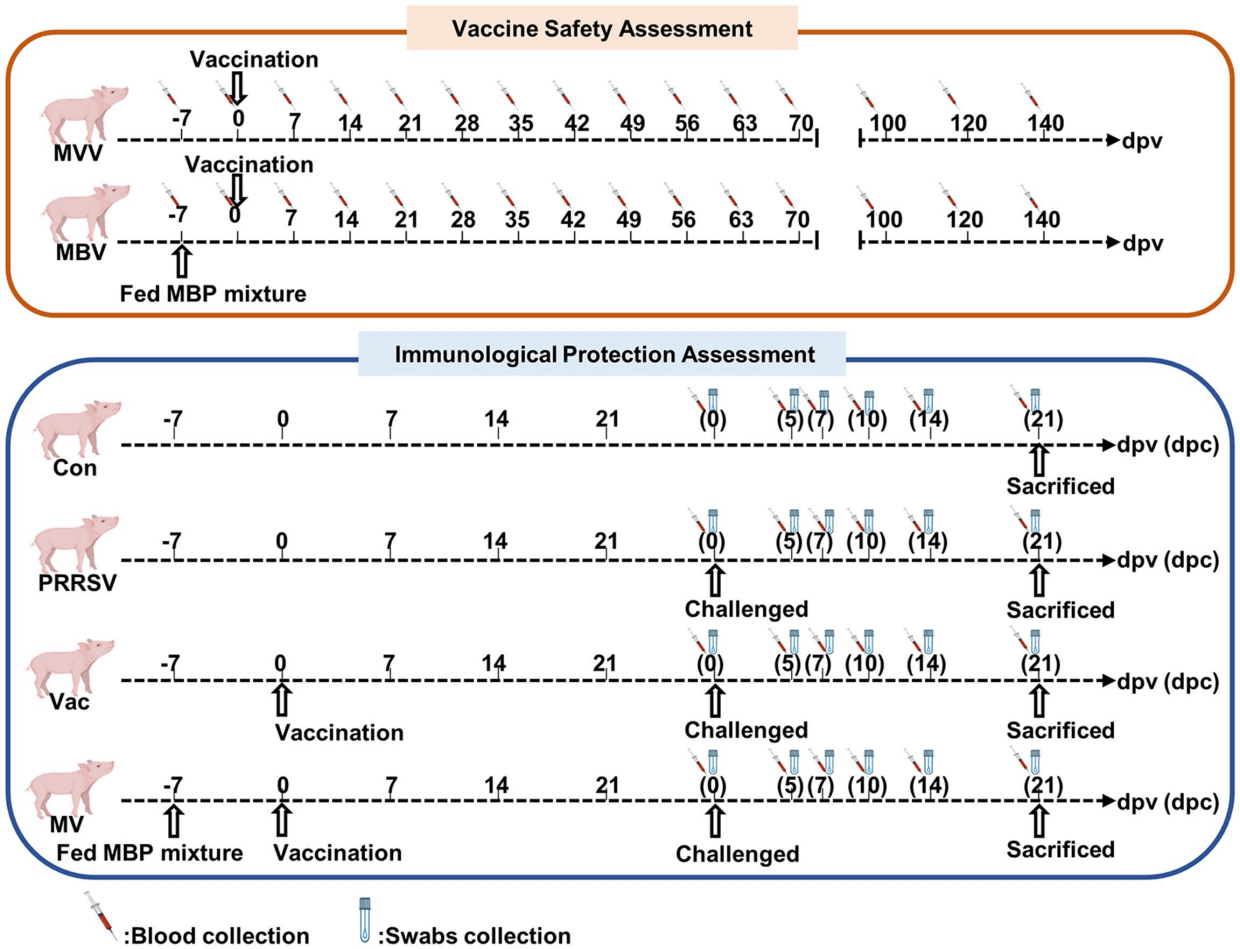
Overview of experiment. −7 represent 7 days prior to vaccine immunization, at which point piglets were supplemented with 0.02% MBP mixture in the standard diet.

The piglets were randomly divided into six groups: a normal control group (Con), a PRRSV control group (PRRSV), a vaccine-only group (MVV), a group treated with vaccine and challenged with HuN4 (Vac), a group treated with MBP combined with vaccine (MBV), and a group treated with MBP-vaccine and challenged with HuN4 (MV). All piglets were fed a standard diet. The MBV and MV groups received supplementation with 0.02% MBP mixture from 7 days before vaccination (−7) to 28 days post-vaccination (dpv). The Vac, MVV, MBV, and MV groups were intramuscularly injected with the live attenuated HuN4-F112 vaccine (batch 2,020,006; Harbin Weike Biotechnology Co., Ltd.) at 0 dpv. At 28 dpv [0 days post-challenge (dpc)], all piglets, except those in the Con, MVV, and MBV groups, were challenged with HP-PRRSV HuN4-F2 (containing 3 × 10^4.5^ TCID_50_) and sacrificed at 21 dpc. Experimental procedures are depicted in Figure 1 and Table 1.

**Table 1:** Summary of experimental groups and schedule of *in vivo* experiments.

Group	Con	MVV	MBV	PRRSV	Vac	MV
*N*	5	5	5	5	5	5
Treatment	–	MLV	MLV and MBP	–	MLV	MLV and MBP
Vaccine	–	0 dpv	0 dpv	–	0 dpv	0 dpv
MBP	–	–	−7-28 dpv	–	–	−7-28 dpv
Challenge	–	–	–	28 dpv	28 dpv	28 dpv
Viremia/viral shedding/immunity factors	−7, 0, 7, 14, 21, 28 dpv and 5, 7,10,14,21 dpc	−7, 0, 7, 14, 21, 28 dpv	−7, 0, 7, 14, 21, 28 dpv	5, 7, 10, 14, 21 dpc	5, 7, 10, 14, 21 dpc	5, 7, 10, 14, 21 dpc
Flow cytometry assay	−7, 0, 14, 28 dpv	−7, 0, 14, 28 dpv	−7, 0, 14, 28 dpv	–	–	–
Traditional Chinese Medicine Ingredient Analysis	28 dpv	-	28 dpv	–	–	–
Rectal temperature/Clinical score	0–21 dpc	–	–	0–21 dpc	0–21 dpc	0–21 dpc
Ab detection	–	7–140 dpv	7–140 dpv	5, 7, 10, 14, 21 dpc	5, 7, 10, 14, 21 dpc	5, 7, 10, 14, 21 dpc
Kill	21 dpc	–	–	21 dpc	21 dpc	21 dpc

### 2.6 Sample collection

Blood samples were collected from the anterior vein to measure immunological factors, neutralizing antibodies, and PRRSV viral load. Additionally, a portion of the blood samples was collected at 28 dpv and used to analyze the presence of traditional Chinese medicine active ingredients. Nasal, oral, and anal swab samples were collected simultaneously to assess viral shedding and measure PRRSV viral load using quantitative RT-PCR. Biochemical estimates of PGE2, IFN-α, IL-6, and IL-1β in blood samples were conducted using ELISA kits across all experimental groups. At 21 dpc, tissue samples were taken from the lymph nodes, tonsils, spleen, lungs, liver, heart, kidneys, stomach, colon, and brain. A portion of the tissue was fixed in 4% paraformaldehyde for pathological analysis, and another portion was stored at – 80°C. For viral load testing, 1 g of tissue samples, 200 μL swab samples, and 200 μL serum samples were used for RNA extraction to measure the viral load in the various samples. The experimental set up and histopathological scoring are presented in Tables 1, 2, respectively.

**Table 2:** Gross and histopathological scoring.

Concept	Severity (scoring)			
	None	Mild (1)	Moderate (2)	Severe (3)
**Macroscopy**				
Oedema	Normal	Recovering quickly from pressure	Moderate oedema recovers longer after compression	Organs are tense and shiny and do not recover for a long time when pressed
Congestion/hemorrhage	Normal	Mild congestion or active hyperemia, diffuse or patchy distributed in parenchyma. No haemorrhages	Multifocal to coalescent randomly distributed petechiae and purpurae. Variable degree of congestion or active hyperemia	Multifocal to coalescent random and interlobulillar distributed extensive hemorrhages (ecchymoses). Variable degree of congestion or active hyperemia
**Histopathology**				
Congestion/hemorrhage	Normal	Capillary hyperemia with minimal to mild multifocal vasculopathy and mild diffuse capillary hyperemia	Mild to moderate angiectasia with mild to moderate multifocal vasculopathy, and mild to moderate diffuse capillary hyperemia	Marked angiectasia with moderate diffuse vasculopathy, mild multifocal vascular necrosis and hemorrhages
Inflammatory infiltrates	Normal	Mild multifocal interstitial mononuclear inflammation	Moderate multifocal interstitial mononuclear inflammation	Marked multifocal (extensive) interstitial mononuclear inflammation
Necrosis	Normal	Destruction of the cellular structure of individual tissues	Destruction of a limited number of cellular structures	Poorly organised, with extensive destruction of cellular structures

### 2.7 Clinical evaluation

Average weight gain (WG) was calculated from 0 to 28 dpv and 0 to 21 dpc, respectively. Daily rectal temperature and clinical signs were recorded from 0–21 dpc. The clinical scores were calculated as previously described (Table 3). Observations were conducted twice daily: once in the morning from 9:00 to 10:00 and once in the afternoon from 14:00 to 15:00. These observations were systematically recorded to ensure consistency and accuracy.

**Table 3:** Criteria for evaluation of clinical signs following HP-PRRSV challenge.

Clinical symptom	Score			
	0	1	2	3
Temperature	Normal	Mild fever (40°C < T < 40.5°C)	High fever (40.5°C < T < 41°C)	High fever (>41°C)
Breathing	Normal (<40/min)	Tachypnea (40–60/min)	Tachypnea (60–80/min) and dyspnea	Tachypnea (greater than 80/min) and severe dyspnea (panting, breathing through open mouth)
Behavior	Active	Apathetic but responding to stimulation	Apathetic even when stimulated	Prostration
Appetite	Normal	Eats less than normal	Not eating	–
Ears	Normal	Redness and heat	Purple/Blue	–
Other symptom (conjunctivitis, coughing, sneezing, vomiting, diarrhea, shivering)	None	One sign presented	Two signs presented	Three or more signs presented

### 2.8 Serum neutralization assay

Sera from piglets were heat-inactivated at 56°C for 30 min before conducting serum neutralization (SN) assays. Then, DMEM was used as the diluent to prepare serial twofold dilutions of each serum sample. Subsequently, suspensions containing 100 TCID_50_ of PRRSV per 100 μL were formulated, and then 100 μL of the suspension was added to each serum dilution. And then, the serum-viral mixtures were incubated at 37°C for 1 h. Subsequently, the plates were further incubated at 37°C in a humidified atmosphere with 5% CO_2_ for 7 days and monitored daily for cytopathic effects (CPE). Finally, the Reed-Muench method was used to calculate the neutralization titer of the serum. Each dilution was replicated four times.

### 2.9 Quantitative real-time PCR

The standard working curve of PRRSV was created using a series of plasmid standards (10^2^–10^9^ copies). Briefly, total RNA was extracted using a Simple P Virus DNA/RNA Extraction Kit. Next, reverse transcription of total RNA into cDNA was done using a Reverse Transcription Kit. Then, TaqMan-based real-time fluorescence quantitative RT-PCR was used to detect RNA copies of PRRSV (15). Quantitative RT-PCR assays were performed using Agilent MX3000P QPCR equipment. All results were obtained from at least three replicates.

### 2.10 Flow cytometry assay

Flow cytometry (Bricyte E6, Shenzhen, China) was used to analyze the immunity of piglets by quantifying T-cell subsets in the peripheral blood. Three-color phenotyping using fluorochrome-conjugated monoclonal antibodies (Southern Biotech, United States) for CD4^+^ and CD8^+^ T cells was conducted for the immunophenotyping of lymphocytes. Samples were processed and evaluated. The ratios of CD3^+^, CD4^+^, CD8^+^, and CD4^+^/CD8^+^, were calculated to assess the health status of the immune system.

### 2.11 Statistical analysis

Data obtained were analyzed using SPSS 17.0 statistical software (IBM, United States), and results were presented as mean ± standard deviation (SD). The Student’s t-test and One-way analysis of variance (ANOVA) was used to analyze group differences. *p* > 0.05 was considered as the level of statistical significance. Pictures were analyzed using GraphPad Prism 8.0.2 software.

## 3 Results

### 3.1 MBP reduces the duration of viremia

**Figure 2:**
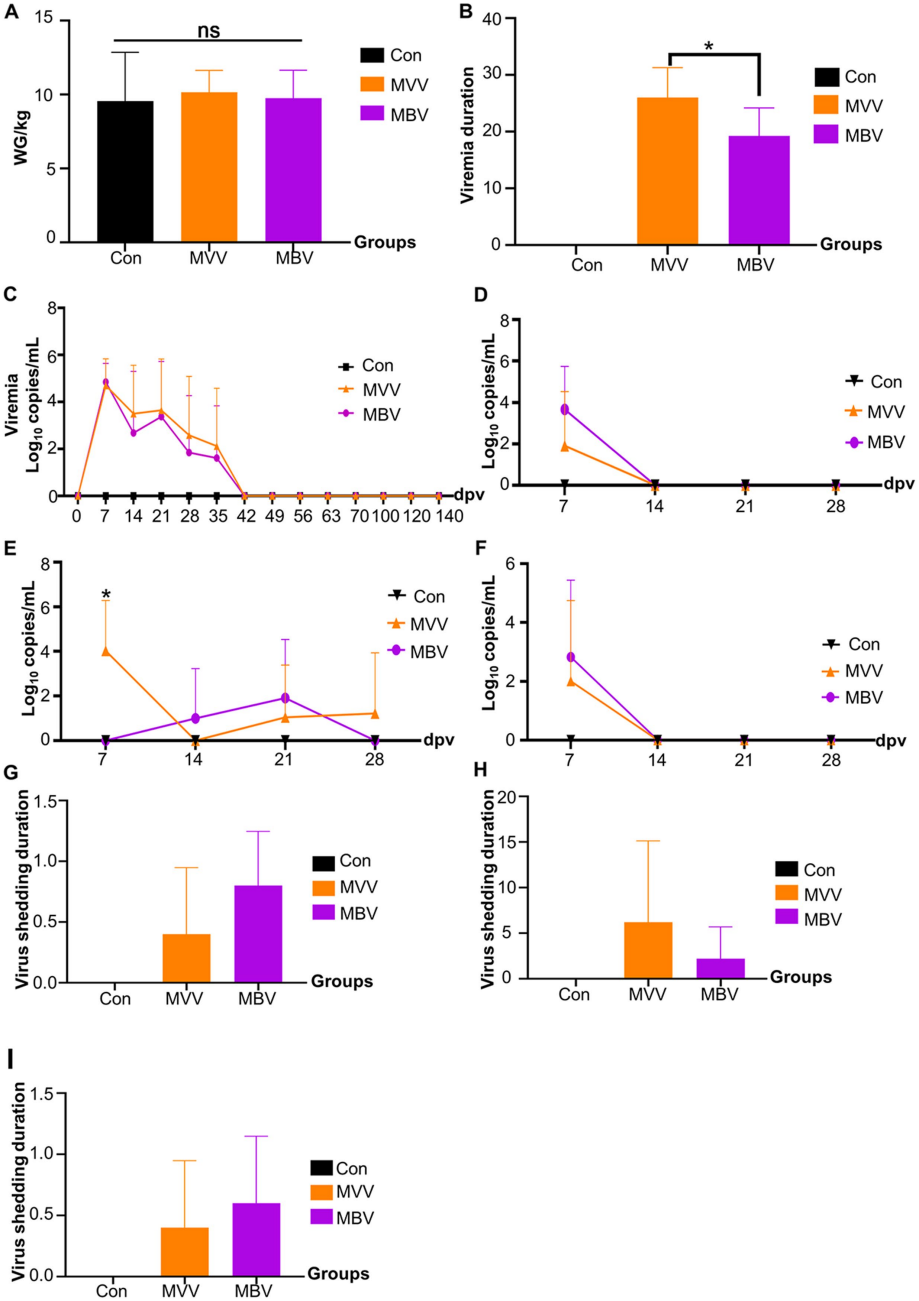
The effect of MBP on vaccine immunity was assessed through various parameters. **(A)** Weight change in piglets before and after vaccination, **(B)** duration of viremia, measured from the first to the last detection of PRRSV ORF7 in serum, **(C)** levels of PRRSV ORF7 copies in serum detected using qRT-PCR, **(D)** expression levels of PRRSV ORF7 copies in nasal swabs, **(E)** oral swabs, and **(F)** anal swabs, also detected using qRT-PCR, and **(G-I)** virus shedding duration in nasal, oral, and anal swabs. Data were analyzed using ANOVA and are presented as mean ± SD. The asterisk (*) indicates significant differences between the MVV group and the MBV group (*p < 0.05).

The average weight gain (WG) from 0–28 dpv was 9.55 kg, 10.16 kg, and 9.76 kg for Con, MVV, and MBV groups, respectively, with no significant differences observed, suggesting that MBP does not significantly affect the productive performance of vaccinated piglets. Given that pigs inoculated with MLV shed viruses, we investigated viremia and viral shedding post-vaccination with HuN4-F112. The results indicated that the combination of MBP and Vac significantly reduced the duration of viremia, although it had no significant effect on viral load (Figure 2B), thereby enhancing vaccine safety without interfering with HuN4-F112 immunogenicity.

### 3.2 MBP enhances cellular immunity

**Figure 3:**
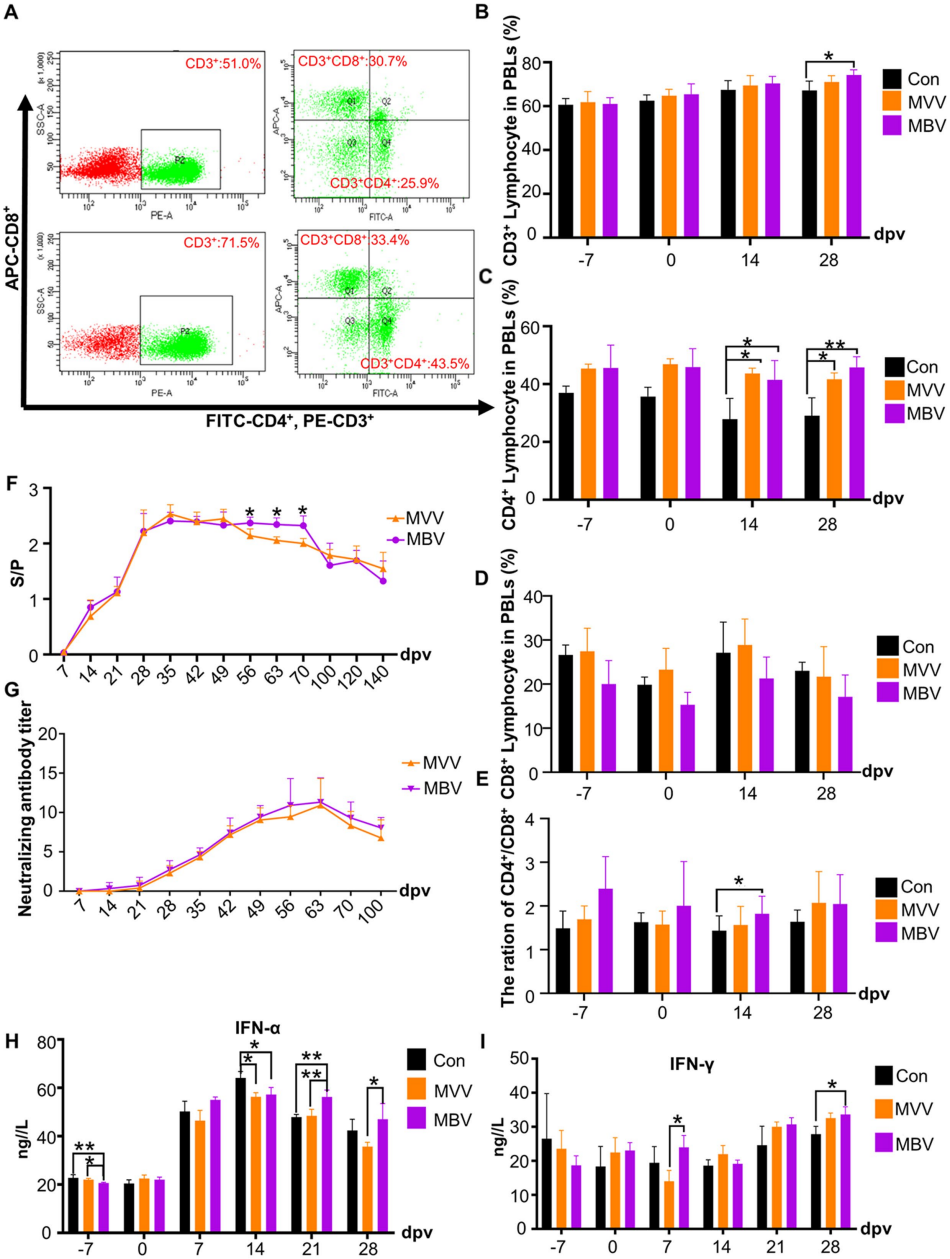
Effects of MBP on piglet immunity. **(A)** Flow cytometry analysis of T lymphocyte subsets in peripheral blood. Average percentages of CD3^+^ **(B)**, CD4^+^ **(C)**, CD8^+^ **(D)**, and the CD4^+^/CD8^+^ ratio **(E)** in lymphocytes are presented as mean ± SD. **(F)** Serum anti-PRRSV antibody levels, with an S/P ratio of ≥0.4 considered antibody positive. **(G)** Neutralizing antibody titer. **(H)** Serum IFN-α levels. **(I)** Serum IFN-γ levels. Except for the data in **F,G**, which were analyzed using the Student’s t-test, all other data were analyzed using ANOVA. Data are presented as mean ± SD. The asterisk (*) indicates significant differences among groups (*p < 0.05).

Previous experiments indicated that MBP has immune-potentiating effects (14). Thus, we used flow cytometry to determine CD3^+^, CD4^+^, and CD8^+^ T cell subpopulations (Figure 3A) to assess MBP’s impact on cellular immunity. At 28 dpv, the MBV group showed a significant increase in CD3^+^ T subpopulations compared to the Con group (*p* < 0.05), with no difference compared to the MVV group (Figure 3B). Notably, pigs immunized with HuN4-F112 exhibited significantly higher CD4^+^ T subpopulations at 14 and 28 dpv in both MVV and MBV groups compared to the Con group (*p* < 0.05) (Figure 3C). CD8^+^T subpopulation generation remained unaffected (Figure 3D). Additionally, the CD4+/ CD8+ T subpopulation ratio in MBV group was significantly higher at 14 dpv compared to the Con group (*p* < 0.05) (Figure 3E). These results confirmed our earlier claim that using MBP in combination with vaccine acts as a potential immunomodulating agent against PRRSV, thus highlighting the need for further research to understand how this combination works at a molecular level.

### 3.3 MBP combined with MLV enhances immunity

In Figure 3F, we observed a sustained increase in antibody levels in the peripheral blood of both the MVV and MBV groups from 28 to 70 (dpv). Importantly, antibody levels were significantly higher in the MBV group than in the MVV group at 56, 63, and 70 dpv (*p* < 0.05), indicating an enhanced and more prolonged antibody response in the MBV group. However, there were no significant differences between the MVV and MBV groups in neutralizing antibody (NA) titers.

In addition, we assessed the levels of two crucial cytokines in immune system activation and antiviral responses, IFN-γ and IFN-α. The MBV group exhibited significantly higher levels of IFN-α at 21 and 28 dpv (Figure 3H) and IFN-γ at 7 dpv (Figure 3I) compared to the MVV group. Furthermore, the MBV group showed significantly elevated IFN-γ levels compared to the Con group at 28 dpv.

### 3.4 MBP alleviates PRRSV-induced clinical symptoms

**Figure 4:**
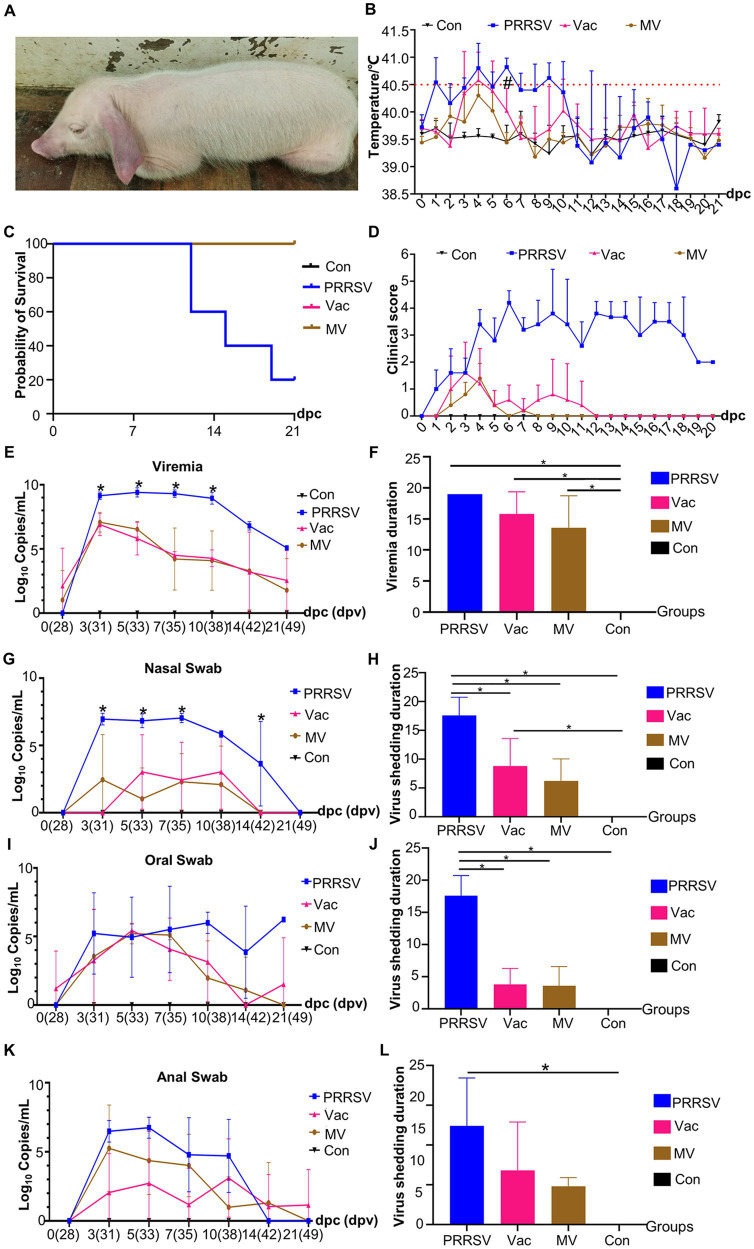
MBP alleviates PRRSV symptoms and disease progression. **(A)** A representative pig from the PRRSV group showing symptoms of PRRSV onset. **(B)** Rectal temperatures of pigs in each group (n = 5), with temperatures exceeding 40.5°C considered as fever (dotted line). **(C)** Survival curves of piglets infected with HP-PRRSV HuN4 in each group. **(D)** Clinical sign scores in piglets from different groups. Quantitative PCR analysis of ORF7 gene expression at different time points was conducted to assess PRRSV viral load in blood samples **(E)**, nasal swabs **(G)**, oral swabs **(I)**, and anal swabs **(K)**. Asterisks (*) denote significant differences between the PRRSV group, the Vac group, and the MV group (*p < 0.05). Persistence of viremia **(F)** and duration of viral shedding via nasal **(H)**, oral **(J)**, and anal swabs **(L)**. Data were analyzed using ANOVA. Asterisks (*) indicate significant differences among groups (*p < 0.05), and hash symbols (#) denote significant differences between the Vac group and the MV group (*^#^p* < 0.05).

Following the observed immune-enhancing effect of MBP, we speculated that combining MBP with vaccines would alleviate clinical symptoms induced by HP-PRRSV. The persistent presence of typical clinical symptoms, including high fever, coughing, loss of appetite, diarrhea, conjunctivitis, and blue ears, was observed in the PRRSV group throughout the experiment (Figure 4A). The PRRSV group had rectal temperatures exceeding 40°C from 1 to 14 dpc following the HP-PRRSV challenge (Figure 4B). From 1 to 12 dpc, the rectal temperature of the Vac group was higher than 40.5°C, with several pigs reaching temperatures greater than 41°C. Despite these observations, no deaths were observed. The MV group had a rectal temperature ranging between 39.5°C and 40.5°C from 1 to 20 dpc, with no pig deaths observed (Figure 4C). Notably, at 6 dpc, the Vac group exhibited a significantly higher rectal temperature than the MV group. More clinical signs were observed in the PRRSV group than in the Vac and MV groups (Figure 4D). In addition, the duration of clinical symptoms decreased from 20 dpc in the PRRSV group to 12 dpc in the Vac group and 8 dpc in the MV group (Figure 4D). Despite this, no significant difference in the duration of clinical symptoms was observed between the Vac group and the MV group throughout the experiment. The clinical scores of the PRRSV group were significantly (*p* < 0.05) higher than those of the Vac and MV groups. And the Con group remained clinically normal throughout the study (Figure 4D).

### 3.5 MBP did not inhibit post-challenge viremia or reduce the duration

We assessed viremia among groups post-HP-PRRSV challenge, noting viremia in all groups except the Con group. Viremia persisted in all pigs in the PRRSV group until death (Figure 4E). The presence of the PRRSV ORF7 gene was observed in pigs in the PRRSV group at 7, 14, and 21 dpc, with a decrease in the number of viremic pigs over time in the Vac and MV groups. The mean duration of viremia was 15.8 days in the Vac group and 13.6 days in the MV group (Figure 4F). However, there was no significant difference in PRRSV ORF7 copies between the Vac and MV groups at any time point (Figure 4E).

We also analyzed viral shedding post-HuN4 challenge, noting the shortest duration from the nasal orifice: 17.6d (PRRSV), 3.8d (Vac), and 3.6d (MV) (Figures 4G,H). However, oral shedding lasted longer: 17.6d (PRRSV), 8.8d (Vac), and 6.2d (MV) (Figures 4I,J), while anal shedding was 12.4d (PRRSV), 6.8d (Vac), and 4.8d (MV) (Figures 4K,L). Viral shedding peaked between 3 and 10 dpc, with the PRRSV group showing the highest loads (Figures 4G,I,K). Specifically, the PRRSV group demonstrated peak shedding of up to 10^7.79^ copies (3 dpc) from nasal swabs, 10^7.53^ copies (5 dpc) from anal swabs, and 10^7.58^ copies (7 dpc) from oral swabs. The PRRSV group consistently showed higher ORF7 copies than the Vac and MV groups, with no significant difference between the Vac and MV groups.

### 3.6 Combination therapy reduces tonsillar viral load and lung damage

**Figure 5:**
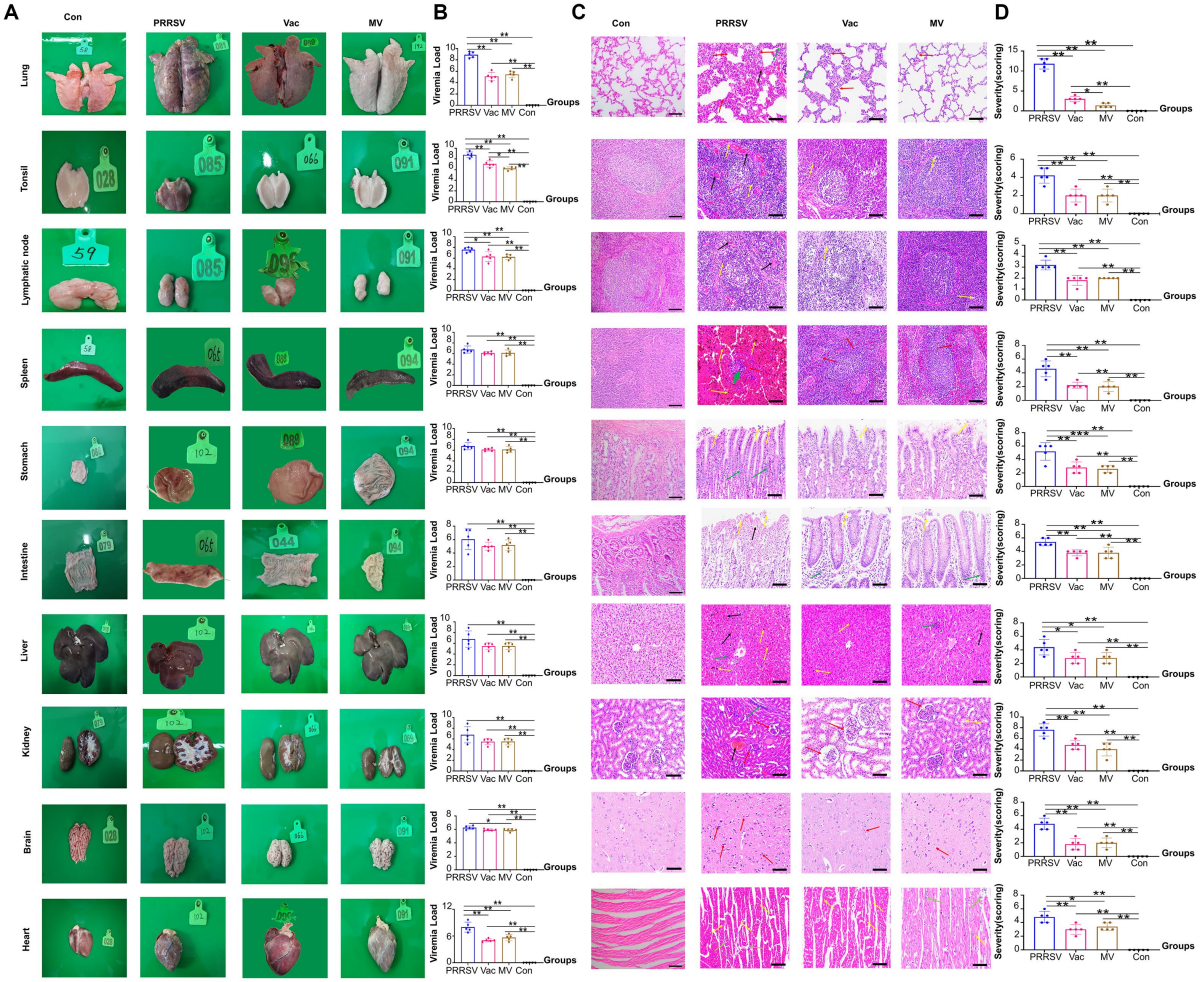
Pathological changes and viral loads in organs of pigs. **(A)** Appearance of organs from the PRRSV, Vac, and MV groups. **(B)** Quantitative PCR analysis of ORF7 gene expression for PRRSV viral load in various organs. **(C)** Histopathological observation of hematoxylin–eosin (H&E)-stained sections showing pathological changes, indicated by arrows. **(D)** Overall histopathological scores for corresponding organs (5 fields per pig; five pigs per group). Data were analyzed using ANOVA. Asterisks (*) indicate significant differences among groups (*p < 0.05; **p < 0.01).

To assess organ damage and vaccine efficacy, we examined the organs of deceased pigs from each group. Immunization with HuN4-F112 reduced hemorrhagic necrotic patches and tissue damage caused by PRRSV compared to the PRRSV group (Figure 5A). QPCR analysis showed higher ORF7 copies in organs of the PRRSV group than in the Vac and MV groups (Figure 5B). Notably, the MV group had significantly lower viral loads in tonsils compared to the Vac group (Figure 5B).

Expanded alveolar septa and reduced alveolar number (red arrows) were observed in interstitial pneumonia, while capillary expansion and hyperemia (black arrows) were observed in the lungs of pigs in the PRRSV group. Then, disrupted structures with widespread cell necrosis (yellow arrows) were observed in the tonsils and lymph nodes. Indistinct medullary areas (red arrows), with cell necrosis and tissue pigmentation (yellow arrows), were observed in the spleen. Subsequently, severe damage and inflammatory cell infiltration (green arrows) were observed in the stomach and intestinal epithelium. The liver had significant pigmentation and infiltration of inflammatory cells (yellow arrows), but no connective tissues were present (Figure 5C). Furthermore, glomerular atrophy and capillary congestion (red arrows) were observed in the kidneys. Moreover, disrupted histological structure with glial cell proliferation (red arrows) was shown in the brain. The cardiomyocyte nuclei exhibited compaction, lysis, or disappearance (yellow arrows) alongside inflammatory infiltration. Furthermore, greater organ damage was observed in the histopathological evaluations in the PRRSV group compared to the Vac and MV groups (Figure 5D).

### 3.7 Combination therapy inhibits inflammation, boosts immunity

**Figure 6:**
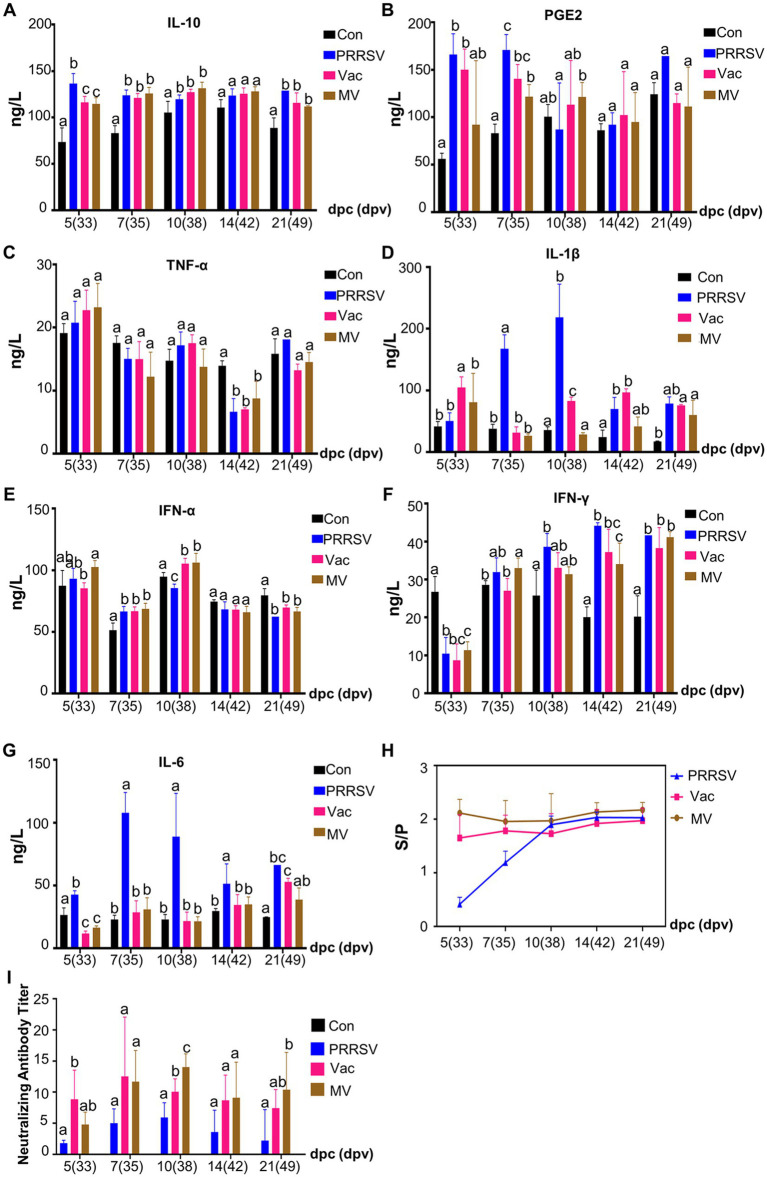
MBP suppresses inflammatory factors and extends the duration of high antibody levels. Serum levels of IL-10 **(A)**, PGE2 **(B)**, TNF-α **(C)**, IL-1β **(D)**, IFN-α **(E)**, IFN-γ **(F)**, and IL-6 **(G)** were quantified using ELISA in each group. The levels of anti-PRRSV antibodies **(H)** and neutralizing antibodies titer **(I)** in serum samples were also measured. Data were analyzed using ANOVA and presented as mean ± SD (*n* = 5). Different lowercase letters indicate significant differences (*p* < 0.05) in multiple-range analysis among the groups.

Cytokines play a crucial role in mediating immune responses, and their dysregulation can lead to organ damage (16). Our study revealed that the PRRSV challenge significantly increased cytokine expression, including IL-10 (Figure 6A), PGE2 (Figure 6B), TNF-α (Figure 6C), IL-1β (Figure 6D), IFN-α (Figure 6E) and IFN-γ (Figure 6F), which remained elevated throughout the experiment. Despite higher IL-10 levels in the PRRSV group, pro-inflammatory cytokines remained unchanged, indicating PRRSV-induced immune dysfunction. Our research showed that PGE2, IL-6, and IL-1β levels increased progressively in the PRRSV-infected group from 5 dpc. However, IL-10 helped normalize these levels in the Vac and MV groups. Interestingly, at 10 dpc, the Vac group had higher IL-1β levels than the MV group, suggesting MBP’s potential to reduce IL-1β-induced injury and expedite recovery.

Innate and cellular immunity, notably influenced by IFN-α and IFN-γ, play crucial roles in inhibiting pathogen life cycles. However, despite significant increases in serum levels of these cytokines following the PRRSV challenge, they failed to alleviate clinical symptoms or organ damage in the PRRSV group (Figures 6E,F). This reveals the insufficiency of relying solely on innate and cellular immunity to combat the pathogen and suggests potential disorders in overall immunity. MBP demonstrated its potential in enhancing innate and cellular immunity, as evidenced by significantly higher levels of IFN-α and IFN-γ in the MV group at 5 and 7 dpc (Figures 6E,F), respectively, compared to the Vac group.

The humoral immune response of piglets was assessed by measuring PRRSV-specific antibodies and NA using an IDEXX ELISA kit and SN assays (Figures 6H,I). PRRSV-specific antibodies were detected in all PRRSV-challenged piglets from 5 dpc (Figure 6H), indicating infection alone induced PRRSV-specific antibody production. However, in the early period of challenge (5 and 7 dpc), antibody levels were significantly higher in the Vac and MV groups than in the PRRSV group, highlighting the efficacy of the HuN4-F112 vaccine in inducing an antibody response. SN assay results at 5 dpc (Figure 6I) showed higher NA titers in the Vac and MV groups compared to the PRRSV group, with titers of 1:8 and 1:5, respectively, while titers in the PRRSV group were < 1:2. NA titers peaked at 1:10 in the Vac group at 7 dpc, while the MV and PRRSV groups reached peaks of 1:14 and 1:6 at 10 dpc, respectively. Notably, at 10 dpc, NA titers in the MV group were higher than those in the Vac group (1:8) (Figure 6I), indicating the role of MBP in maintaining elevated NA levels in pig serum for an extended period.

Overall, our findings demonstrated that the oral application of MBP combined with vaccination significantly inhibited the inflammatory cytokine storm triggered by the PRRSV challenge in growing pigs. This strategy effectively suppressed the expression of pro-inflammatory cytokines, repaired damaged lungs, improved cellular and innate immunity, and prolonged high levels of NA. Which likely contributed to the mitigation of clinical symptoms and disease progression.

## 4 Discussion

Building on an earlier study regarding MBP’s immune-enhancing activity, we embarked on more comprehensive research to study the mechanism by which MBP helps improve immune function following PRRS MLV-induced immunodeficiency. Additionally, we assessed the protective effects of combining MBP with vaccines against the HP-PRRSV HuN4 challenge and analyzed the active ingredients in MBP that contributed to its efficacy. Through these investigations, we revealed that MBP is a potential immunopotentiator that can attenuate PRRSV challenges and help mitigate vaccine limitations.

As shown in Figure 2C, no significant difference was seen in plasma viral load between the MVV and MBV groups post-vaccination, indicating MBP’s inclusion did not affect MLV immunogenicity. PRRSV MLV vaccines are known for viral shedding post-vaccination, confirmed by nasal, oral, and anal swabs collected up to 28 days after vaccination. Our analysis of viral shedding revealed prolonged shedding in oral swabs (Figure 2). This could likely be due to PRRSV replication in the tonsils, a primary site, and subsequent dissemination into oral fluid (17). Previous studies have suggested oral fluid as a viable alternative to serum for PRRSV detection in pigs (18, 19). Still, our findings revealed the importance of collecting blood and oral fluid samples for a more accurate assessment of PRRSV presence. Despite no significant difference in shedding duration from oral swabs, there was a notable reduction in the duration of viremia in the MBV group post-vaccination compared to MVV. Given MBP’s absence of direct anti-PRRSV activities, we speculated on its efficacy in modulating the body’s immune response, boosting disease resistance, and potentially averting persistent PRRSV infection.

T lymphocytes play a critical role in immune responses, with the CD3^+^ molecule used to identify total T lymphocyte counts (20). Therefore, we examined CD4^+^ and CD8^+^ T cell subsets and the CD4^+^/CD8^+^ ratio in piglets from MVV and MBV groups (Figure 3). There were no significant differences in the percentages of CD4^+^ and CD8^+^ T cells or the CD4^+^/CD8^+^ ratio between the MVV and MBV groups over the study period. However, at 28 dpv, the MBV group showed a significantly higher percentage of CD3^+^ T cells than the Con group, indicating an increase in total T lymphocyte count due to MBP presence. Furthermore, the MBV group exhibited significantly higher levels of IFN-γ at 28 dpv, consistent with CD3^+^ assay findings, suggesting MBP’s role in enhancing cellular immunity. Subsequently, we evaluated humoral immunity by comparing the MVV and MBV groups using the S/*p* value and NA titer. Although there was no significant difference in NA titers between the MVV and MBV groups over time, the MBV group consistently showed slightly higher NA titers. Additionally, during the mid-phase of the experiment (56–70 dpv), the S/*p* values in the MBV group were higher than those in the MVV group, implying a positive regulatory effect of MBP on humoral immunity (Figure 3).

Following infection with HP-PRRSV, pigs commonly experience a high fever, a symptom that remains challenging to control even with vaccination (20). In our study, we observed that piglets challenged with the HuN4 strain exhibited prolonged high fever more frequently in the Vac group compared to the MV group (Figure 4). High fever can be induced by various cytokines, such as IL-1β, IL-6, and TNF-α, which possess inflammatory and thermogenic properties. These cytokines trigger PGE2 production through different pathways, ultimately leading to fever (21, 22). HP-PRRSV induces a robust inflammatory response, resulting in an “inflammatory cytokine storm,” contributing to its heightened pathogenicity. While IL-1β positively impacts host antiviral activity, excessive expression of IL-1β can trigger a “cytokine storm” that leads to a toxic inflammatory response, exacerbating viral pathogenicity (23). In this study, IL-1β and IL-6 levels peaked at 7–10 dpc in the PRRSV group and 5 dpc in the Vac and MV groups. Notably, at 5 and 10 dpc, IL-1β levels were significantly lower in the MV group compared to the Vac group. Previous studies have linked HP-PRRSV-induced fever to IL-1β (24), potentially explaining the lower incidence and duration of fever observed in the MV group compared to the Vac group.

From previous studies, it was established that elevated levels of pro-inflammatory cytokines are closely associated with persistent infection with histopathological evidence as observed in cases of PRRSV (25). Further investigations by Li and others have demonstrated that HP-PRRSV exacerbates inflammation, resulting in tissue and organ damage (26). Hence, we used an improved version of the method developed by Galindo-Cardie to quantitatively assess the macroscopy and histopathology of PRRSV-infected pigs (27). In our study, we administered MBP orally, observed its beneficial effects on clinical symptoms and lung injury, and compared the Vac and the MV groups (Figures 4, 5). The positive outcome observed for the MV group was attributed to the ability of MBP to inhibit the inflammatory response. The anti-inflammatory and pharmacological effects of MBP are due to the combined action of its multiple active ingredients (28, 29).

Given the broad tissue tropism of PRRSV, we examined viral loads in various organs. We found a significant reduction in tonsillar viral load in the MV group compared to the Vac group (Figure 5). Tonsils, rich in macrophages (30), are prime targets for PRRSV infection, facilitating prolonged viral presence and potential transmission via oral and nasal secretions (31). Our findings suggested that MBP intervention accelerated the clearance of PRRSV in tonsils, potentially inhibiting PRRSV spread. Additionally, we investigated nasal and oral viral shedding duration post-infection in MBP-treated piglets (Figure 4). While differences were not significant, the MBP group showed a trend towards reduced shedding, revealing an inhibition of viral transmission via oral and nasal routes.

Antibody responses to PRRSV were once considered ineffective or harmful due to antibody-dependent potentiation effects. However, recent studies highlight the protective role of neutralizing antibodies (NA) against PRRSV, revealing their importance in protection against viral infections (32, 33). Animals with low NA titers experience viremia, while higher titers can inhibit viremia but may not prevent viral replication and transmission (34). Our study showed NA titers peaked in the Vac and MV groups at 7 and 10 dpc, respectively. However, viremia persisted. Young pigs with more susceptible macrophages may require higher NA levels for protection (34). Although the SN assay on Marc-145 cells may not fully represent *in vivo* conditions, our results demonstrated higher NA levels in the MV group (Figure 6), suggesting an enhanced immune response. Besides, NA, IFN-α, and IFN-γ also play significant roles in preventing PRRSV infection (35). MBP intervention contributes to the elevation of IFN-α and IFN-γ levels (Figure 6), highlighting its role in enhancing innate and cellular immunity against PRRSV, thereby protecting piglets post-infection.

## 5 Conclusion

Finally, in comparison to vaccination alone, the oral administration of modified Bazhen powder (MBP) and MLV vaccination elicited a prompt, comprehensive, and effective protective immunological response, as seen by elevated levels of antibody titers and rapid viremia clearance. In addition, following the HP-PRRSV challenge, it significantly ameliorated lung injury scores, mitigated the severity of lung damage, and decreased tonsil virus loads. The oral administration of MBP also significantly decreased TNF-α, IL-6, and IL-1β levels, demonstrating its potential to attenuate the inflammatory storm that follows PRRSV infection. These results showed that MBP has the potential to function as an oral immunomodulator, improving vaccination safety and offering significant protection against PRRSV infection in pigs.

## Data availability statement

The data presented in the study are deposited in the Figshare repository, accession number 10.6084/m9.figshare.26413546 (https://figshare.com/articles/dataset/Untitled_Item/26413546).

## Ethics statement

The animal study was approved by Experimental Animal Ethical Committee of Northeast Agricultural University (NEAUEC202303113). The study was conducted in accordance with the local legislation and institutional requirements.

## Author contributions

HC: Writing – review & editing, Methodology, Visualization, Writing – original draft. YW: Methodology, Visualization, Writing – original draft, Writing – review & editing. WC: Writing – review & editing, Software. GH: Software, Writing – review & editing. B-OG: Software, Writing – review & editing. YLiu: Software, Writing – review & editing, Resources. CD: Writing – review & editing, Investigation, Project administration. ZZ: Writing – review & editing, Methodology, Supervision, Validation. YLi: Writing – review & editing, Conceptualization, Data curation, Formal analysis, Funding acquisition, Investigation.
