# Corrigendum: Protection efficacy and the safety of the synergy between modified Bazhen powder and PRRSV modified-live virus vaccine against HP-PRRSV in piglets

**DOI:** 10.3389/fvets.2024.1498769

**Published:** 2024-10-28

**Authors:** Hua Chai, Yanru Wei, Wenguang Chen, Guorui Han, Bello-Onaghise Godspower, Yanyan Liu, Chunliu Dong, Zhiyun Zhang, Yanhua Li

**Affiliations:** ^1^College of Veterinary Medicine, Northeast Agricultural University, Harbin, China; ^2^Department of Animal Science, Faculty of Agriculture, University of Benin, Benin City, Nigeria

**Keywords:** HP-PRRSV HuN4, modified live vaccine, modified Bazhen powder, efficacy, safety, synergy

In the published article, there was an error in the author list, and Professors Chunliu Dong and Zhiyun Zhang were erroneously excluded as corresponding authors. The corrected author list appears below:

Hua Chai^1†^, Yanru Wei^1†^, Wenguang Chen^1^, Guorui Han^1^, Bello-Onaghise Godspower^1, 2^, Yanyan Liu^1^, Chunliu Dong^1*^, Zhiyun Zhang^1*^ and Yanhua Li^1*^

^1^College of Veterinary Medicine, Northeast Agricultural University, Harbin, China

^2^Department of Animal Science, Faculty of Agriculture, University of Benin, Benin City, Nigeria

^†^These authors have contributed equally to this work and share first authorship.

^*^ **Correspondence**:

Chunliu Dong

dcliu@neau.edu.cn

Zhiyun Zhang

zhangzhiyun@neau.edu.cn

Yanhua Li

liyanhua@neau.edu.cn

The authors apologize for this error and state that this does not change the scientific conclusions of the article in any way. The original article has been updated.

