# Supplementary material for: Protection efficacy and the safety of the synergy between modified Bazhen powder and PRRSV modified-live virus vaccine against HP-PRRSV in piglets
Source: Front Vet Sci. 2024 Aug 5;11:1436426. doi: 10.3389/fvets.2024.1436426 (PMC11331794; doi:10.3389/fvets.2024.1436426)
Supplement: Supplementary file 1 [file Data_Sheet_1.docx]

Supplementary Material

# Traditional Chinese Medicine ingredient analysis

The Vanquish Horizon UHPLC system (Thermo Fisher Scientific) equipped with a Water UPLC BEH C18 column (2.1 × 100 mm × 1.7 µm) was utilized for the LC-MS/MS analysis of blood samples. This was followed by the determination of bioactive ingredients of MBP absorbed by the blood using a quadrupole time of flight and tandem mass spectrometer (UHPLC-Q-TOF-MS/MS).

Total ion current (TIC) chromatograms of blood samples in the positive and negative ion modes from the Vac group and the MV group were conducted and UPLC-Q-TOF-MS was used to determine the amount of components by the absorbed blood. A total of twenty-three compounds were characterized (Fig. S1 and S2).

The 23 components were subjected to analysis using the following online databases: TCMID <http://www.megabionet.org/tcmid/>） (1), TCMSP（<https://old.tcmsp-e.com/tcmsp.php>） (2) and BATMAN-TCM（<http://bionet.ncpsb.org.cn/batman-tcm/>） (3), along with a review of the literatures, which confirmed that Betaine, Soyasaponin Ⅰ, Ononin, 5-O-Methylvisammioside and 18-Beta-glycyrrhetinic acid were present in the formulated herbal mixture (2023.08.19).


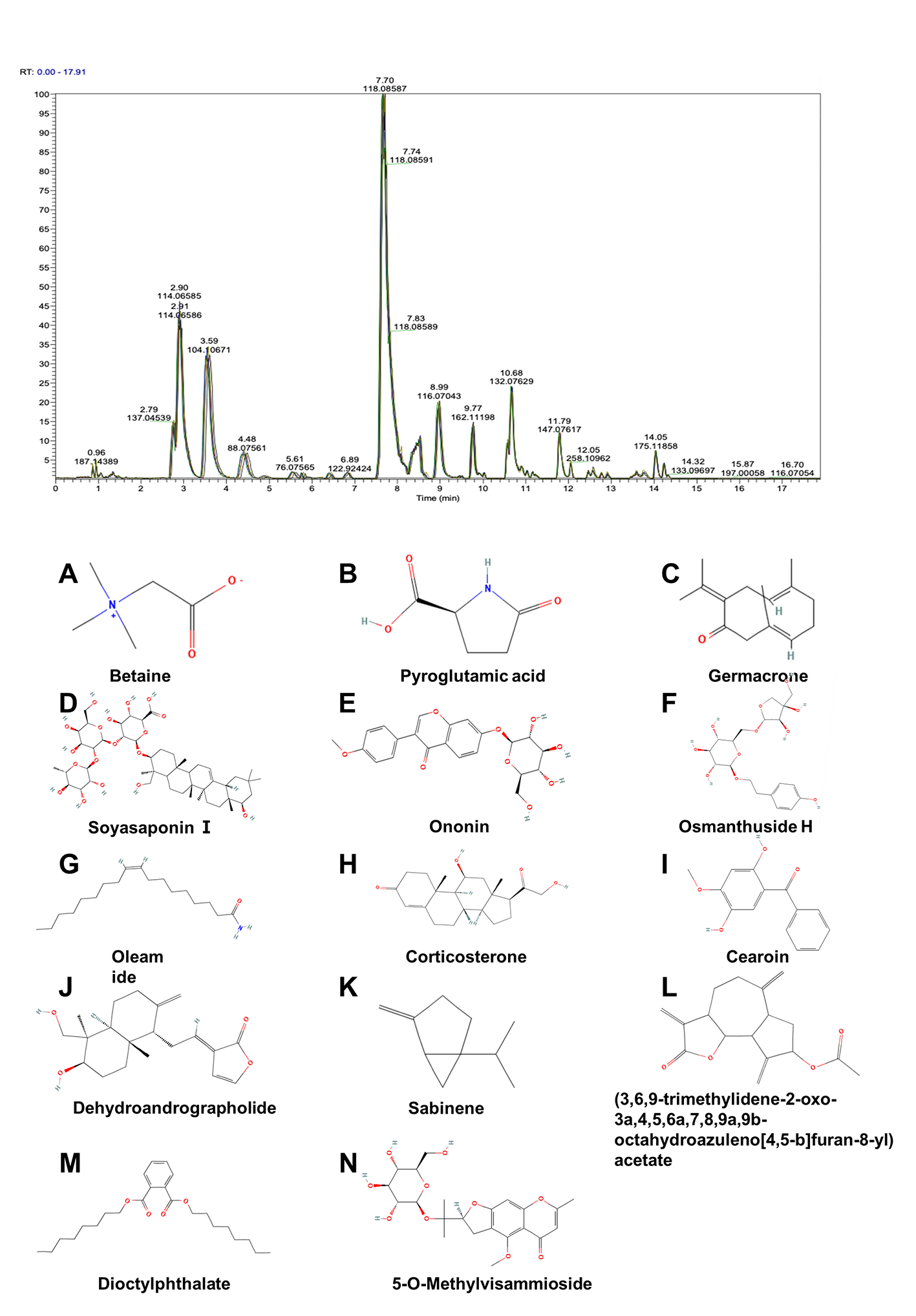


**Supplementary Figure 1.** **Chromatogram and chemical structure of MBP components in piglet plasma after MBP dietary supplementation, analyzed in positive mode**. **(A–N)** Components absorbed by the blood include: Betaine, Pyroglutamic acid, Germacrone, Soyasaponin Bb, Oleamide, Ononin, Sabinene, Osmanthuside H, Corticosterone, Cearoin, Dehydroandrographolide, Dioctylphthalate, 5-O-Methylvisammioside, (3,6,9-trimethylidene-2-oxo-3a,4,5,6a,7,8,9a,9b-octahydroazuleno[4,5-b]furan-8-yl) acetate and 2-Chloro-DL-Phenylalanine.


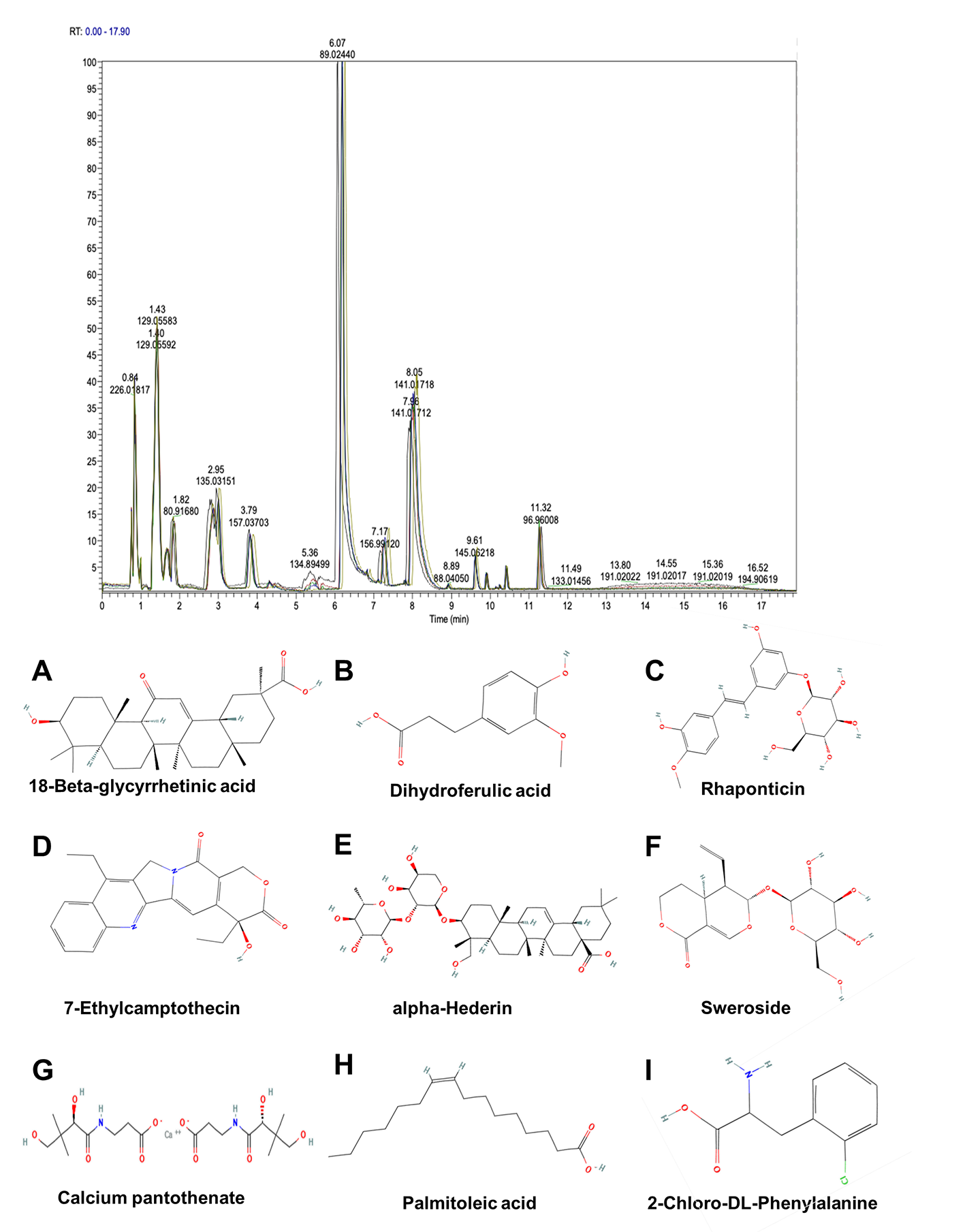


**Supplementary Figure 2.Chromatogram and chemical structure of MBP components in piglet plasma after MBP dietary supplementation, analyzed in negative mode**. **(A–I)**. Components absorbed by the blood include: 18-Beta-glycyrrhetinic acid, Dihydroferulic acid, Rhaponticin, 7-Ethylcamptothecin, alpha-Hederin, Sweroside, Calcium pantothenate, Palmitoleic acid and 2-Chloro-DL-Phenylalanine.

**Reference**

1. Xue R, Fang Z, Zhang M, Yi Z, Wen C, Shi T. Tcmid: Traditional Chinese Medicine Integrative Database for Herb Molecular Mechanism Analysis. *Nucleic Acids Research* (2013) (D1):D1089-95.

2. Ru J, Li P, Wang J, Zhou W, Li B, Huang C, et al. Tcmsp: A Database of Systems Pharmacology for Drug Discovery from Herbal Medicines. *J Cheminform* (2014) 6(1):13.

3. Liu Z, Guo F, Wang Y, Li C, Zhang X, Li H, et al. Batman-Tcm: A Bioinformatics Analysis Tool for Molecular Mechanism of Traditional Chinese Medicine. *Scientific Reports* (2016) 6:21146.
